# C-reactive protein and procalcitonin in patients with DRESS syndrome

**DOI:** 10.1186/2045-7022-4-S3-P12

**Published:** 2014-07-18

**Authors:** Anne Taegtmeyer, Alexandra Raetz Bravo, Barbara Zimmermanns, Evangelia Liakoni, Stephan Kraehenbuehl, Manuel Haschke

**Affiliations:** 1 Basel University Hospital, Division of Clinical Pharmacology & Toxicology, Switzerland; 2 Basel University Hospital, Regional Pharmacovigilance Centre, Switzerland

## Background

C-reactive protein (CRP) and procalcitonin (PCT) are acute phase proteins whose concentrations are used to guide the management of bacterial infections. The CRP- and PCT-response in DRESS syndrome is not clearly documented in the literature. Elevated CRP or PCT may lead to management dilemas regarding discontinuation or switching of antibiotic therapy in these patients.

## Methods

We examined cases of DRESS syndrome which were reported to our pharmacovigilance centre 2008-2013 and cases reported in the literature during the same period for which CRP and/or PCT were available. Peak values were recorded whenever possible. The extent of CRP and PCT elevation in cases where DRESS syndrome was not due to antibiotics was also studied as these cases were less likely to have underlying infection as a potential confounding factor.

## Results

34 cases were identified (18 from pharmacovigilance reports). Antiepileptic agents and antibiotics were judged to be causal in 11 cases each (8 beta-lactams), sulfasalazine in 3 and allopurinol and antiviral agents in 2 cases each. The remaining cases were combinations of antibiotics and another drug (n=3), 1 case of metamizole in combination with pantoprazole and a case where the causative drug was not given. CRP was available for 33 (97%) cases and ranged from 28 - 420mg/l (median 98, normal <10mg/l). PCT was available in 9 (26%) cases and ranged from 0.4 - 15.5ng/ml (median 2, normal <0.1ng/ml). In cases where no antibiotics were involved (n=16), CRP concentrations ranged from 28 - 301 mg/l and PCT concentrations from 0.4 - 15.5ng/ml (n=7). Mean (± SD) CRP concentrations were 146 ± 117mg/l and 115 ± 84mg/l in cases where antibiotics were and were not suspected as the cause of DRESS, respectively (p=0.2). For PCT these figures were 4.96 ± 3.83ng/ml and 2.03 ± 0.9ng/ml, respectively. By way of comparison, median CRP values of 42mg/l, 65mg/l and 102mg/l have been reported in patients with sepsis, severe sepsis and septic shock, respectively. For PCT these values were 0.19ng/ml, 0.46ng/ml and 6.5ng/ml, respectively.

## Conclusion

CRP and PCT are elevated in DRESS syndrome and can reach levels normally seen in acute bacterial infections, sepsis or even septic shock. This is of importance for making the correct diagnosis, particularly as the management of these conditions differs widely. Further analyses are required to determine the correlation of these biomarkers with the severity and outcome of DRESS syndrome.

